# Sleep Does Not Promote Solving Classical Insight Problems and Magic Tricks

**DOI:** 10.3389/fnhum.2018.00072

**Published:** 2018-02-26

**Authors:** Monika Schönauer, Svenja Brodt, Dorothee Pöhlchen, Anja Breßmer, Amory H. Danek, Steffen Gais

**Affiliations:** ^1^Institute of Medical Psychology and Behavioral Neurobiology, University of Tübingen, Tübingen, Germany; ^2^Department of Psychology, Ludwig-Maximilians-Universität München, Munich, Germany; ^3^Division of Neurobiology, Department Biology II, Ludwig-Maximilians-Universität München, Munich, Germany

**Keywords:** information processing, sleep/wake cognition, sleep and memory, problem solving, insight, incubation

## Abstract

During creative problem solving, initial solution attempts often fail because of self-imposed constraints that prevent us from *thinking out of the box*. In order to solve a problem successfully, the problem representation has to be restructured by combining elements of available knowledge in novel and creative ways. It has been suggested that sleep supports the reorganization of memory representations, ultimately aiding problem solving. In this study, we systematically tested the effect of sleep and time on problem solving, using classical insight tasks and magic tricks. Solving these tasks explicitly requires a restructuring of the problem representation and may be accompanied by a subjective feeling of insight. In two sessions, 77 participants had to solve classical insight problems and magic tricks. The two sessions either occurred consecutively or were spaced 3 h apart, with the time in between spent either sleeping or awake. We found that sleep affected neither general solution rates nor the number of solutions accompanied by sudden subjective insight. Our study thus adds to accumulating evidence that sleep does not provide an environment that facilitates the qualitative restructuring of memory representations and enables problem solving.

## Introduction

When facing a difficult problem, we often get the advice to “*let it rest”* or “*sleep on it.”* Indeed, some studies support the view that a period of incubation can subserve problem solving, and it has recently been suggested that sleep may particularly contribute to this psychological function. It is, however, still a matter of debate under which conditions such beneficial effects occur.

When attempting to solve a problem, the individual elements that constitute the task have to be represented in memory and integrated with prior knowledge to arrive at a solution. This process can either be based on analytic reasoning, which leads to a solution in an incremental step-by-step manner, or the solution may emerge as a result of sudden insight (Bowden and Jung-Beeman, 2003; Gilhooly, 2016; Llewellyn, 2016). Even though it is difficult to pinpoint the phenomenon of insight and to define exact criteria of insight tasks (Chronicle et al., 2004; Helie and Sun, 2010), it seems clear that insight involves a change in the representation of a cognitive concept (Ohlsson, 1992; Kounios and Beeman, 2014).

Importantly, insight requires recombining memory and knowledge elements in an innovative, non-obvious fashion by flexibly switching between different associations (Kounios and Beeman, 2014). Öllinger et al. (2014) propose a multi-stage model of representational change during insight problem solving that divides the process into distinct, but dependent stages. First, different solutions are attempted (1) which lead to consistent failure (2). This creates an impasse (3). Only by restructuring the problem representation (4) can insight (Aha!) occur (5) and a solution (6) be reached (Ohlsson, 1992; Knoblich et al., 1999). Accordingly, reaching an impasse increases the likelihood of a later insight experience and subsequent solution of the problem. The better participants could reach the boundaries of their initial solution space, the easier it was for them to overcome them in a second step. When a first problem representation is created and no solution can be found within the assumed problem space, the original problem representation has to be modified by representational change. This change requires relaxing self-imposed constraints on applicable knowledge (Ohlsson, 1992; Knoblich et al., 1999). Other conceptualizations of insight problem solving follow a similar logic, but emphasize that the problem space is initially kept as small as possible and will only modified if no satisfactory progress can be made (Ormerod et al., 2013). Together, both theories share the view that constraints on applicable knowledge are modified during insight problem solving.

Phenomenologically, solving a complex problem is often accompanied by an Aha! experience, meaning that the solution arises in an unexpected, sudden manner, is experienced as obviously correct, and elicits a positive emotional response (Kaplan and Simon, 1990; Gick and Lockhart, 1995; Bowden et al., 2005; Subramaniam et al., 2009; Topolinski and Reber, 2010; Danek et al., 2014; Salvi et al., 2016; Danek and Wiley, 2017). Furthermore, the solution is often preceded by a period of feeling stuck and might therefore be driven by unconscious processing (Bowden et al., 2005). Indeed, qualitative and quantitative reviews have come to the conclusion that a period during which a problem is set aside, termed incubation, is beneficial for insight problem solving (Dodds et al., 2003; Sio and Ormerod, 2009).

Many studies investigate insight problem solving with the remote associates test (RAT) and observe that a period of incubation improves performance (Mednick et al., 1964; Dodds et al., 2002; Vul and Pashler, 2007). According to Gilhooly (2016), these incubation benefits might be facilitated by automated spreading of activation along associative links, coupled with an active but below-threshold goal representation. In line with this assumption, it has been shown that a prime can spark later insight (Cai et al., 2009). Alternatively, activation of neural traces representing unsuccessful solution attempts might decay over a period of incubation, making a relaxation of previous constraints and consequent activation of novel networks more likely (Öllinger et al., 2008).

In principle, sleep offers a period of brain isolation that could have similar benefits as an incubation period in wakefulness. Additionally, sleep might even aid the restructuring of a problem representation by qualitatively transforming memories (Stickgold and Walker, 2013). It has been established that sleep has a positive effect on the stabilization of newly acquired memory content, meaning that it helps to preserve stored information (Gais and Born, 2004; Schabus et al., 2004; Diekelmann and Born, 2010; Rasch and Born, 2013; Schönauer et al., 2014b). The standard model of memory consolidation assumes that by repetitive reactivation of hippocampo-cortical networks during sleep, initially hippocampal-dependent memories become gradually integrated into cortico-cortical networks, which leads to a reorganization of memory representations on the neural level (Frankland and Bontempi, 2005; Takashima et al., 2006; Gais et al., 2007). Whether and under which circumstances this process also entails a qualitative reorganization of the memories is currently under debate (Lewis and Durrant, 2011; Inostroza and Born, 2013; Stickgold and Walker, 2013; Ackermann and Rasch, 2014; Landmann et al., 2014). Arguing in favor of this idea are studies that investigate the integration of newly encoded information into existing knowledge networks in lexical integration tasks (Tamminen and Gaskell, 2013) and the formation of false memories for words representing the gist of actually studied word lists, which are induced by sleep (Payne et al., 2009; Diekelmann et al., 2011).

Additionally, there is ample evidence that sleep can promote the extraction of statistical regularities, for example in probabilistic learning (Durrant et al., 2011) or in transitive inference tasks (Ellenbogen et al., 2007), making access to patterns and rules explicit (Stickgold and Walker, 2013). It is assumed that repetitive reactivation of overlapping memory representations in sleep helps to extract regularities from multiple memories (Djonlagic et al., 2009; Lewis and Durrant, 2011). Wagner et al. (2004) investigated problem solving in a version of the Number Reduction Task that could either be solved analytically or via a hidden short cut rule. Subjects used the short cut during a delayed test more often when they spent the time after the initial confrontation with the task asleep than when they stayed awake, indicating that knowledge of the hidden rules governing the task improved over sleep.

Creative problem solving, contrary to memory integration, concept formation, or rule learning, requires the ability to disintegrate existing concepts in order to recombine memory representations in a novel fashion (Landmann et al., 2014). So far, a beneficial effect of sleep on this kind of problem solving is still being discussed (Monaghan et al., 2015; Landmann et al., 2016; Debarnot et al., 2017) and the scarce evidence yields mixed results (Cai et al., 2009; Sio et al., 2013; Beijamini et al., 2014). To our knowledge, the study by Beijamini et al. (2014) is the only one that investigates the effect of sleep on a problem that requires logical reasoning. Most other sleep studies use the RAT, which is described as an insight problem but actually relies more on associative processing than on the restructuring of a problem representation. In the RAT three seemingly unrelated words (e.g., ‘dust’/‘cereal’/‘fish’) are presented and participants are asked to find the expression that connects those three words (‘bowl’). Apart from a general benefit of incubation, Cai et al. (2009) found that participants with REM sleep between sessions had higher solution rates in the RAT than participants without REM sleep. In contrast, Landmann et al. (2016) did not observe a sleep-related improvement in the closely similar Compound Remote Associates Test, but only better memory retention for previously solved items, supporting a primarily stabilizing role of sleep.

The goal of the present study was to disentangle effects of incubation and sleep on insight problem solving. Four groups of subjects were confronted with a set of classical problem solving tasks consisting of matchstick-algorithms, the nine-dot problem and the eight-coin problem (Danek et al., 2016). Furthermore, participants had to find out how several magic tricks worked, a novel insight paradigm (Danek et al., 2014). So far, studies on sleep and problem solving have not addressed the nature of the solutions that were reached by the participants (Walker et al., 2002; Cai et al., 2009; Beijamini et al., 2014; Monaghan et al., 2015; Landmann et al., 2016). Therefore, to be able to separate sudden solutions, which are typical for insight problem solving, from analytical step-by-step problem solving, we also collected qualitative data about the participants’ subjective feeling of sudden insight, and compared qualitative and quantitative performance measures (Danek and Wiley, 2017). We hypothesized that an incubation period would increase insight experiences and overall solution rates. Similar to awake incubation, sleep keeps the brain from conscious processing but may additionally support the restructuring of underlying problem representations. Therefore, we further expected that incubation during sleep would have a larger effect than incubation during wakefulness.

## Materials and Methods

### Participants

A total of 77 subjects (46 females and 31 males, mean age 23.8 ± 3.4 years [M ± SD]) participated in this experiment in one of four groups. The sample size was based on previous studies (e.g., Cai et al., 2009; Sio et al., 2013), which found significant effects with average group sizes of 12–15 subjects. All subjects were healthy and reported a regular circadian rhythm with a sleep duration of 6–10 h. There were no extreme morning or evening types in the sample, as assessed by the Munich Chronotype Questionnaire (Roenneberg et al., 2007). Subjects were selected to report no problems falling asleep in unfamiliar environments as well as being able to sleep during the afternoon. They had no long-distance flights within the 6 weeks preceding the experiment. Starting two nights before the experiment, participants filled in a sleep log, giving information about their bed time and when they woke up. The two mornings before the experiment, participants were instructed to wake up 1 h earlier than usual. This mild sleep restriction was applied to facilitate falling asleep in an afternoon nap (Schönauer et al., 2014b; Studte et al., 2015). Sleep recordings from two participants were defective, but online EEG surveillance assured that those participants were able to fall asleep and their data were still included in group-level statistics. Data from nine participants were missing for the magic tricks because of technical problems with the audio recording. Nine participants reported prior knowledge of the nine-dot problem and were excluded from the analysis of the classical insight tasks.

### Testing Material

#### Magic Tricks

In the first task, participants were shown 10 short (19.5 s ± 10.5 s [M ± SD]) video clips of magic tricks and instructed to figure out the rationale behind them (“Please try to find out how the trick works!”). Tricks were performed by a professional magician and recorded in a standardized setting. Stimulus development and the experimental rationale behind using magic tricks as insight tasks are described in detail elsewhere (Danek et al., 2014). Each trick was based on one single technique (e.g., misdirection, sleight-of-hand) and had one specific effect (e.g., disappearance, telekinesis; see **Table 1**). Furthermore, the tricks were difficult to see through, with a solution rate of less than 10% after the first presentation (Danek et al., 2014). Instructions for the task as well as the video clips were presented on a 23-inch computer screen. Participants’ answers with their suggested explanation of the magic trick were recorded by the computer.

**Table 1:** The 10 magic tricks.

Magic trick	Effect	Description
Vanishing coin	Vanish	Out of three coins, one vanishes.
Rubik’s cube	Transformation	An unsolved Rubik’s cube is solved after being tossed into the air.
Ketchup bottle	Vanish	A ketchup bottle is put in a bag and disappears.
Match through match	Penetration	One matchstick wanders through another one without breaking it.
Salt	Vanish	Salt is poured in the fist from where it disappears.
Torn and restored card	Restoration	A card is ripped in pieces and restored.
Water to ice	Transformation	Water is poured into a mug and transformed into an ice cube.
Floating bun	Telekinesis (Levitation)	A bun is covered by a napkin and starts to float.
Bowling ball	Topological impossibility (size)	A large bowling ball is carried in a thin suitcase.
Shuffled/Unshuffled	Telekinesis	Cards are seen mixed face-up/face-down, before all facing the same way (as if they had turned over by themselves).

#### Matchstick Arithmetic Task

**FIGURE 1:**
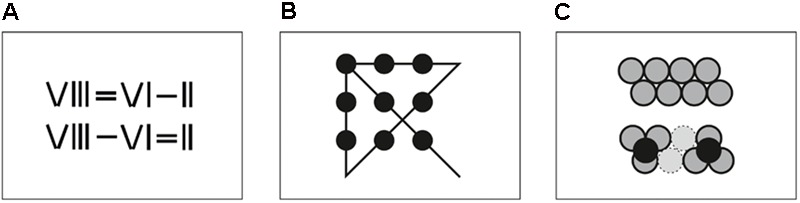
Schematic illustration of the three tasks used to investigate the process of insight problem solving. **(A)** Matchstick arithmetics: Here the mathematical expression depicted in the first line has to be transformed to a correct mathematical expression by moving matchsticks around. **(B)** Nine-dot problem: In this task, participants have to connect the nine dots with four straight lines without lifting the pen from the paper. **(C)** Eight-coin problem: Here, the eight coins in the first row have to be moved in a way that each coin touches exactly three other coins.

In matchstick arithmetic tasks (see **Figure 1A**), participants are presented with incorrect arithmetic equations, with roman numerals and operators depicted as combinations of matchsticks. The correct solution requires moving one single matchstick to correct the equation. The problem employed in this study was taken from a group of previously used matchstick arithmetic tasks (Öllinger et al., 2008). In this case “VIII = VI – II” has to be transformed to “VIII – VI = II” by moving one matchstick from the equal sign on top of the minus sign. The task was depicted on a sheet of paper as well as with real matchsticks. Participants moved the matchsticks, but could return to the start configuration at any time.

#### Nine-Dot Problem

This classical task (Scheerer, 1963) requires 9 dots in a 3 × 3 grid to be connected with 4 straight lines, without lifting the pen from the paper (see **Figure 1B**). In the correct solution, the lines extend beyond the boundaries of the grid (Kershaw and Ohlsson, 2004). Participants were presented with the dots (each with one centimeter diameter) depicted on a sheet of paper and drew the lines directly on the sheet. Material was provided for three drawing attempts.

#### Eight-Coin Problem

In the eight-coin problem the coins are arranged in two slightly shifted rows of four coins each (see **Figure 1C**) (Ormerod et al., 2002). The subject has to find a constellation where each coin touches exactly three other coins by moving exactly two coins. The solution consists of two clusters of three coins each with a fourth coin resting on top of each cluster. Eight 20-cent coins were spread out on a table. Participants were allowed to touch the coins and move them around during the problem solving process. The initial arrangement of the coins was always visible on a sheet of paper, so that they could get back to the start configuration at any time.

### General Procedure

Experimental procedures were approved by the Ethics Committee of the Department of Psychology at Ludwig-Maximilians-Universität München, and participants gave written informed consent. In a 3-h daytime sleep vs. wake × incubation vs. no incubation design, four groups of participants worked on the two types of problem solving tasks described above. Subjects were assigned to the groups randomly as they were recruited. It was, however, assured that all groups had a final sample size of 19 or 20 subjects. The tasks were presented in a standardized fashion in a soundproof chamber with no experimenter present. Instructions were given exclusively on the computer screen or on paper. Spoken solutions were recorded in the magic tricks task. The classical problem solving tasks were solved by the subject with the material provided (matchsticks, paper, coins) and left in the experimental chamber. Accordingly, no interaction between subjects and experimenters was necessary during the problem solving tasks. This was done to prevent Rosenthal effects of experimenter expectancy. This is particularly important in insight tasks, because even subtle and inadvertent hints or differences in instructions can strongly influence solution probability.

**FIGURE 2:**
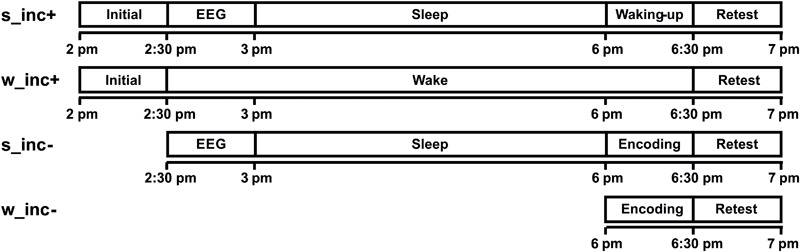
Study design. All subjects stayed in the laboratory from 2 pm to 7 pm. The two experimental groups sleep incubation (s_inc+) and wake incubation (w_inc+) experienced a 3-h incubation period between initial encoding and retest. Both control groups (s_inc- and w_inc-) did not experience an incubation period, but the sleep no incubation group (s_inc–) slept for 3 h before encoding and retest. EEG: application of EEG electrodes.

Participants attempted to solve the problems first during a short initial encoding phase and then again during a second solution attempt during a longer retesting phase. Two experimental groups were subjected to a 4h-incubation interval spent asleep (sleep incubation, s_inc+) or awake (wake incubation, w_inc+) between initial problem encoding and retest phase. Two control groups spent an interval asleep (sleep no incubation, s_inc-) or awake (wake no incubation, w_inc-) before initial encoding. This first solution attempt was then immediately followed by the retest phase (**Figure 2**). Three hour sleep periods started 30 min after the s_inc+ group encoded the task. Sleep was followed by a 30-min wake-up phase before further testing. Sleep was surveilled by EEG recording. Participants in the wake groups stayed in the laboratory under the supervision of the experimenters and were not allowed to sleep or eat. To prevent them from thinking about the problems, they spent the time reading magazines and playing board games with the experimenters. Videos, instructions and experimental timing were implemented in the Matlab toolbox Cogent 2000.

The first solution attempt served as a period to encode the problem representations. For the magic tricks, participants were given standardized audiotaped instructions and viewed each of the randomly presented video clips once. After each video, participants were given 30 s to consider a solution. Then they could provide their possible solution by speaking into a microphone. Subsequently, the three classical insight tasks were brought into the room by the experimenter. After reading the instructions, participants had at maximum 1 min per problem to come up with a solution. The problems were always presented in the same order (matchstick arithmetic, nine-dot problem, eight-coin problem). After each magic trick and problem, participants were asked to indicate whether they had already known the solution beforehand.

During the second solution attempt, each of the ten magic tricks was shown twice, regardless of whether it had been solved during the initial phase. The procedure was similar to the initial phase: after each magic trick, participants had the possibility to attempt a solution. Classical insight tasks that had been solved during the initial phase were not presented again in the retesting phase. For the remaining problems, participants were given up to 5 min to find a solution. After every magic trick and problem, participants stated whether the solution came to their minds in a step-wise or in a sudden manner and whether the solution had already appeared during the incubation period.

### Polysomnography

Sleep data was recorded in the sleep lab using electroencephalography (EEG) at C3 and C4 electrode positions according to the 10–20 system. In addition, bipolar horizontal and vertical electrooculography (EOG) and electromyography (EMG) of the chin was recorded. Data was sampled at a rate of 250 Hz. Reference and ground electrodes were placed on the nose and on the forehead, respectively. All channels were notch-filtered at 50 Hz. Additionally, EEG channels were filtered between 0.5 and 30 Hz, a 10 Hz low pass filter was applied to the EOG channels and a 25 Hz high pass filter was applied to the EMG. All recordings were scored offline in 30 s epochs by two independent raters and disagreements were resolved by a third rater according to the standard criteria by Kales and Rechtschaffen (1968).

### Statistical Analysis

We calculated solution rates by dividing the number of correct answers by the number of previously unknown and unsolved tasks, separately for the magic tricks and the classical insight problems. Solutions for the magic tricks were rated by two independent raters. An answer was counted as correct, when both raters agreed. Following guidelines by Viera and Garrett (2005), inter-observer agreement was high (Cohen’s K initial phase = 0.93; Cohen’s K retest phase = 0.87). Analyses of variances were done in SPSS 21 and were based on ranked dependent variables when the Levene-Test indicated lacking homogeneity of variances. Additionally, we conducted non-parametric *X*^2^ tests when values of the dependent variable were dichotomous. Significance was reached at a two-sided level of α = 0.05. All values are given as mean ± standard error of the mean (M ± SEM) if not indicated otherwise.

## Results

### Magic Tricks

**FIGURE 3:**
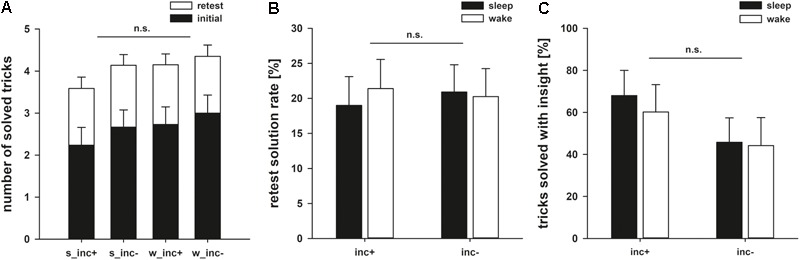
**(A)** Number of solved tricks after the initial and retest phase. **(B)** Retest performance rates for the magic tricks for groups with incubation (inc+) and without incubation (inc–). **(C)** Percentages of solved tricks that were solved with insight instead of analytical step-by-step problem solving. Here, we observe a numerical difference between the experimental and control groups, with incubation increasing the number of tricks solved with insight.

None of the participants knew the solution to any of the 10 magic tricks beforehand. Participants solved 2.66 ± 0.21 magic tricks in the initial phase. Importantly, groups did not differ in their initial performance as shown by a 2 × 2 ANOVA with the factors following incubation (*F*_1,64_ = 0.734, *p* = 0.395, $\eta_{p}^{2}$ = 0.011) and following state (sleep/wake: *F*_1,64_ = 0.479, *p* = 0.492, $\eta_{p}^{2}$ = 0.007; interaction: *F*_1,64_ = 0.194, *p* = 0.661, $\eta_{p}^{2}$ = 0.003; **Figure 3**).

In the retest phase, participants solved an additional 1.40 ± 0.13 tricks. The ANOVA on the rate of additionally solved tricks showed that neither incubation nor sleep led to better performance (incubation: *F*_1,64_ = 0.008, *p* = 0.930, $\eta_{p}^{2}$ < 0.001; state*: F*_1,64_ = 0.047, *p* = 0.829, $\eta_{p}^{2}$ = 0.001; interaction: *F*_1,64_ = 0.149, *p* = 0.701, $\eta_{p}^{2}$ = 0.002; **Table 2**).

**Table 2:** Performance in the initial and retest phase on the magic tricks.

Group (n)	Number of tricks solved during the initial phase	Additionally solved tricks during the retest phase	Net performance increase (%)	Overall solution rate (%)
s_inc+ (17)	2.24 ± 0.43	1.35 ± 0.27	19.00 ± 4.09	35.88 ± 5.50
s_inc- (15)	2.66 ± 0.41	1.47 ± 0.26	20.94 ± 3.91	41.33 ± 4.96
w_inc+ (19)	2.74 ± 0.42	1.42 ± 0.26	21.64 ± 4.16	41.58 ± 5.48
w_inc- (17)	3.00 ± 0.43	1.35 ± 0.27	20.25 ± 4.01	43.53 ± 5.00

In addition to the quantitative analyses above, we investigated possible qualitative differences of the problem solving process. Of all correct solutions, 54 ± 6.3% were solved with insight (as measured by the subjectively reported suddenness in the emergence of the solution). When specifically looking at those tricks that were solved with insight, we saw that a period of incubation numerically, but non-significantly aided performance in the retest phase (incubation: *F*_1,46_ = 2.305, *p* = 0.136, $\eta_{p}^{2}$ = 0.048; state: *F*_1,46_ = 0.139, *p* = 0.711, $\eta_{p}^{2}$ = 0.003; interaction: *F*_1,46_ = 0.060, *p* = 0.807, $\eta_{p}^{2}$ = 0.001; **Figure 3**).

Sleep groups s_inc+ and s_inc- did not differ in the length of S1 (*t*_34_ = 0.482, *p* = 0.633), S2 (*t*_34_ = 0.629, *p* = 0.533), SWS (*t*_34_ = -1.594, *p* = 0.120), REM sleep (*t*_34_ = -1.459, *p* = 0.154), total sleep time (TST; *t*_34_ = -1.409, *p* = 0.168), or wake (*t*_34_ = 0.065, *p* = 0.227; **Table 3**).

**Table 3:** Minutes (M ± SD) spent in sleep stages.

Group (n)	Sleep stage					
	S1	S2	SWS	REM	TST^a^	Wake
s_inc+ (19)	33.03 ± 22.31	41.10 ± 20.56	37.13 ± 22.36	10.13 ± 12.86	121.39 ± 27.75	28.58 ± 27.70
s_inc- (17)	29.44 ± 22.29	36.79 ± 20.48	50.32 ± 27.27	16.67 ± 14.06	133.24 ± 21.93	18.88 ± 17.94

*^*a*^TST, total sleep time.*

We did not observe any significant correlation between the time spent in specific sleep stages and performance on the magic tricks (see **Table 4**). As REM sleep has been suggested to play a specific role in creative problem solving (Cai et al., 2009), we also tested whether participants with REM sleep (*n* = 7; 20.6 ± 4.7% of magic tricks solved) performed better than did participants without REM sleep (*n* = 9; 19.8 ± 6.7% of magic tricks solved). Again, we did not see an effect of REM sleep on performance (*t*_14_ = 0.091, *p* = 0.929).

**Table 4:** Correlations between memory performance measures and specific sleep stages in s_inc+.

		S1	S2	SWS	REM	TST^a^	Wake
Retest solution rates of the magic tricks						
	*r*	0.178	-0.191	-0.065	0.003	-0.060	-0.008
	*p*	*0.510*	*0.479*	*0.810*	*0.990*	*0.826*	*0.976*
Percentage of tricks correctly solved with insight						
	*r*	-0.341	0.184	-0.226	0.040	-0.158	0.185
	*p*	*0.277*	*0.568*	*0.480*	*0.901*	*0.625*	*0.565*

*^*a*^TST, total sleep time.*

### Classical Problem Solving Tasks

The three classical problems have, as expected, different levels of difficulty: 45.5 ± 5.7% of participants could already solve the matchstick algorithms after the initial phase. The nine-dot problem and the eight-coin paradigm had initial solution rates of 6.0 ± 2.9% and 8.0 ± 3.1%, respectively (**Table 5**).

**Table 5:** Solution rates (M ± SEM) for the three classical insight tasks in the initial and the retest phase.

	Matchsticks (%)	Nine-dot (%)	Eight-coin (%)
**s_inc+**			
Initial	40.0 ± 11.2	0.0 ± 0.0	5.0 ± 5.0
Retest	66.7 ± 14.2	36.8 ± 11.4	21.1 ± 9.6
**s_inc-**			
Initial	39.0 ± 11.8	9.0 ± 9.1	0.0 ± 0.0
Retest	72.7 ± 14.1	20.0 ± 13.3	33.3 ± 11.4
**w_inc+**			
Initial	48.0 ± 11.2	10 ± 6.6	10.0 ± 6.6
Retest	90.9 ± 9.1	26.3 ± 10.4	42.1 ± 11.6
**w_inc-**			
Initial	56.0 ± 12.1	6.0 ± 5.9	17.0 ± 9.0
Retest	87.5 ± 12.5	31.3 ± 12.0	33.3 ± 12.6

**FIGURE 4:**
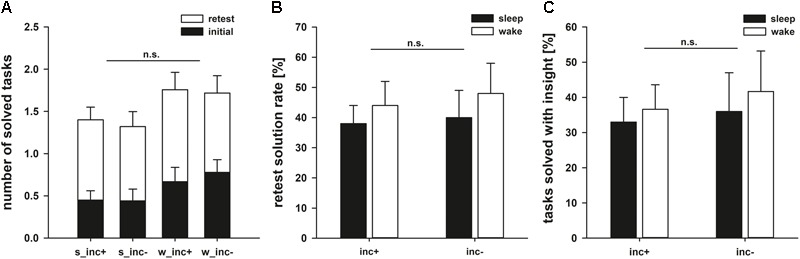
**(A)** Performance after the first (black bars) and the second (white bars) encounters. Note that the maximum number of tricks to be solved was 3. **(B)** Retest solution rates. As the insight tasks were not presented again when they had been solved in the initial phase, 100% represents having solved all remaining insight tasks. **(C)** Percentage of tasks that were solved with insight.

We first assessed whether the four experimental groups differed with regard to solution rates in the initial phase, during which the problem representation was formed. ANOVA revealed neither a main effect of following state (*F*_1,73_ = 2.521, *p* = 0.117, $\eta_{p}^{2}$ = 0.033) nor a main effect of following incubation (*F*_1,73_ = 0.484, *p* = 0.489, $\eta_{p}^{2}$ = 0.007) on solution rates, calculated as the number of solved problems divided by the number of unknown problems. There was also no specific benefit of spending the following incubation period asleep as shown by a non-significant interaction between state and incubation (*F*_1,73_ = 0.039, *p* = 0.845, $\eta_{p}^{2}$ = 0.001; **Figure 4A**).

To answer the question whether incubation or sleep aid creative problem solving, we compared solution rates in the retest phase. Problems that had not yet been solved were presented again, but this time for 5 min instead of one. Again, we neither observed an effect of incubation (*F*_1,73_ = 0.131, *p* = 0.719, $\eta_{p}^{2}$ = 0.002) nor an effect of state (*F*_1,73_ = 0.641, *p* = 0.426, $\eta_{p}^{2}$ = 0.009) on the solution rate. Furthermore, we could not observe the expected interaction between both factors (*F*_1,73_ = 0.006, *p* = 0.939, $\eta_{p}^{2}$ < 0.001; **Figure 4B**). In our study, incubation between two solution attempts therefore does not support problem solving, regardless of whether this time is spent awake or asleep.

Individual insight problems differed largely with regard to difficulty. In addition to looking at the combined performance measure of all three insight tasks, we used the Chi-squared test to investigate each individual problem separately in three additional exploratory analyses. We found no differences regarding retest performance between the four groups in the matchstick arithmetic (*X*^2^ = 2.607, df = 3, *p* = 0.456, *N* = 42), the nine-dot paradigm (*X*^2^ = 1.038, df = 3, *p* = 0.792, *N* = 64) or the eight-coin paradigm (*X*^2^ = 1.947, df = 3, *p* = 0.583, *N* = 71).

We also investigated whether sleep or incubation had an effect on the number of insight experiences. 66% of the correct matchstick arithmetic tasks, 42% of the correct nine-dot problems and 47% of the correct eight-coin problems were achieved with subjectively reported insight. However, there was neither an effect of sleep nor of incubation on the number of insight-related correct solutions (incubation: *F*_1,47_ = 0.188, *p* = 0.667, $\eta_{p}^{2}$ = 0.004; state: *F*_1,47_ = 0.217, *p* = 0.644, $\eta_{p}^{2}$ = 0.005; interaction: *F*_1,47_ = 0.011, *p* = 0.916; $\eta_{p}^{2}$ < 0.001; **Figure 4C**). As shown in **Table 6**, there were no significant correlations between the time spent in different sleep stages and the ability to find solutions to the tasks or the likelihood to solve a problem with insight. When comparing sleep periods with REM sleep (40.0 ± 7.9% of classical problems solved) to sleep without REM sleep (35.2 ± 10.9% of classical problems solved), we also found no difference in retest performance on the classical tasks (*t*_17_ = 0.362, *p* = 0.722).

**Table 6:** Correlations between memory performance measures and specific sleep stages in s_inc+.

		S1	S2	SWS	REM	TST^a^	Wake
Retest solution rates of the classical problems						
	*r*	0.477	-0.307	-0.211	-0.297	-0.151	0.311
	*p*	0.039^∗^	0.201	0.387	0.216	0.537	0.195
Percentage of classical problems correctly solved with insight						
	*r*	-0.002	0.270	-0.226	-0.285	-0.073	0.100
	*p*	0.994	0.396	0.480	0.418	0.823	0.758

**^*a*^*TST, total sleep time. ^∗^Significance thresholds after Bonferroni correction: *p* ≤ 0.008 when correcting for separate tests on sleep stages within each dependent measure, *p* ≤ 0.004 when correcting for all tests in table.*

## Discussion

In this study, we tested the effect of incubation and sleep on solving classical insight problems (Danek et al., 2016) as well as magic tricks (Danek et al., 2014). Previous work has not directly tested whether incubation or sleep affect tasks that critically require a restructuring of the problem representation. Using a daytime sleep versus wake design, we aimed at disentangling effects on both quantitative as well as qualitative measures of insight problem solving. We found that neither an incubation period nor sleep aided the solution of classical problems or magic tricks.

Regarding incubation, we did not find any significant effects on quantitative or qualitative measures of insight problem solving for either of the two tasks. Only numerically, more magic tricks were solved with sudden insight after an incubation period compared to no incubation. Recent meta-analyses report small to medium sized effects for incubation in insight problem solving (Dodds et al., 2003; Sio and Ormerod, 2009; Strick et al., 2011; Gilhooly, 2016). Interestingly, a remarkable number of studies do not, or only partly, report incubation effects (Segal, 2004; Bowden et al., 2005; Vul and Pashler, 2007; Weisberg, 2015). This may indicate that certain tasks profit more from incubation than others. In line with this, Gilhooly (2016) suggests that spreading activation is the main mechanism underlying incubation effects. Indeed, the RAT, which is frequently used to study problem solving (Mednick et al., 1964; Smith and Blankenship, 1991; Dodds et al., 2002; Vul and Pashler, 2007; Sio et al., 2013; Landmann et al., 2016) has been linked to spreading activation (Topolinski and Reber, 2010; Kounios and Beeman, 2014). The tasks we use in the present study, however, require different resources than finding remote associates. Here, the unconscious activation of broad semantic networks will not be sufficient to facilitate problem solving. Instead, the representation of the problem (i.e., the implicit constraints on the solution space in the insight tasks and the limiting presuppositions regarding the magic tricks) has to be restructured in order to successfully solve the task (Danek et al., 2014, 2016; Öllinger et al., 2014). Our results indicate that incubation alone may not support the restructuring of problem representations.

Concerning the role of sleep, it has been suggested that it may support the qualitative restructuring of problem representations that is needed for insight problem solving (Diekelmann and Born, 2010; Stickgold and Walker, 2013). A reactivation of recently encoded material in the hippocampus is assumed to gradually incorporate new material into preexisting cortical mnemonic networks, possibly also forming the basis for a qualitative restructuring of memory representations. It is unclear, whether the reorganization of hippocampal and neocortical memory traces also entails a change in overt behavioral responses (Orban et al., 2006; Deliens et al., 2013; Durrant et al., 2013; Landmann et al., 2014). A couple of studies have investigated the effect of sleep on problem solving ability (Wagner et al., 2004; Cai et al., 2009; Sio et al., 2013; Beijamini et al., 2014; Landmann et al., 2016). Regarding tasks that require a restructuring of mental representations, evidence for a role of sleep is mixed. Recently, Debarnot et al. (2017) only partly replicated an effect of sleep on gaining knowledge of a hidden abstract rule in the above mentioned Number Reduction Task (Wagner et al., 2004). Using the same design as that study, they found a benefit of sleep in young, but not in older participants. Wagner and colleagues also did not replicate the high solution rates after sleep in another study (Verleger et al., 2013). All three studies did not report measures to prevent experimenter expectancy effects. Beijamini et al. (2014) investigated the effect of sleep on a logical reasoning game that requires restructuring and insight. At the point when subjects did not know how to proceed to a higher level, half of them were allowed to sleep for a nap while the other half stayed awake. The nap significantly increased the chance of solving the level on which they had previously been stuck. Unfortunately, performance rates prior to the incubation interval are not shown, which makes interpretation of results more uncertain.

On the other hand, there seems to be stronger evidence for a role of sleep in solving associative tasks. Sio et al. (2013) found in the RAT a benefit of sleep for difficult, but not for easy problems. These difficult problems have weaker direct associations between stimulus and target and require a stronger spread of activation to activate the solution, which is a function that can be supported by sleep (Stickgold et al., 1999). Cai et al. (2009) found that the occurrence of REM sleep increased the chance to find remote associates in the RAT, but only if the correct solution had been primed before sleep. Landmann et al. (2016) did not replicate the finding by Sio et al. (2013), using the similar Compound Remote Associates Test. In their study, a period of sleep did not increase the likelihood of correctly solving previously primed RAT items. All of these findings point toward a sleep-dependent spread of activation that benefits the strengthening of available solutions, but less toward a restructuring of knowledge during sleep. In the present study, solution rates as well as the subjective quality of the solution in the sense of an Aha! experience did not show any effect of sleep. Thus, there is a growing body of evidence showing that sleep does not generally promote the restructuring of newly encoded problems in memory. It may support this process only, e.g., if a spread of associative information or a strengthening of the existing memory representation are sufficient to improve performance. Consequently, our results do not reinforce the notion that sleep-inherent reactivation of newly encoded material and the resulting systems memory consolidation affect problem restructuring and the recombination of knowledge elements required for insightful problem solving.

Performance rates and ratios between insight and non-insight solutions in our experiment are comparable to published studies testing the same paradigms. Tasks were pre-tested to confirm that participants obtained sufficient exposure to solve the presented problems (Ormerod et al., 2002; Danek et al., 2014, 2016; Öllinger et al., 2014). Solution rates in the matchsticks task closely resemble previously reported rates (Schul et al., 2008; Danek et al., 2016). Regarding the eight-coin task, an early study by Ormerod et al. (2002) reports rather low solution rates of below 2%. In recent studies on the eight-coin task, solution rates between 25 and 40% are reported, which are similar to the 35% reported here (Öllinger et al., 2013; Danek et al., 2016).

While we specifically chose the tasks in order to induce a mental fixation, the preparation times might have been too short to induce a strong state of impasse. Instead of interrupting the natural problem solving process, future studies should introduce subjective measures to assess the stage of impasse during problem solving more directly, allowing participants to think about possible solutions until they state to have thoroughly exhausted their ideas. Moreover, it has been shown that incubation time positively correlates with incubation effects (Sio and Ormerod, 2009). Because it has been shown that the length of sleep can impact the observed size of beneficial effects (Diekelmann et al., 2012; Schönauer et al., 2014a) one might argue that our wake and sleep incubation periods were too short for the expected effect to emerge. However, it has been demonstrated that a nap can actually yield comparable effects to a full night of sleep and avoid confounds by circadian rhythm and sleep deprivation of participants (Mednick et al., 2003; Lahl et al., 2008).

The current study used several different problem solving tasks within the same session. It is possible that sleep has only a limited amount of processing capability or that tasks interfered with each other, and that the effect of sleep was therefore diminished or canceled. On the other hand, using a number of different remote associate problems in a RAT task or different memory tasks does not necessarily abolish the effect of sleep (Cai et al., 2009; Schönauer et al., 2015). Rather, we believe that using a number of different problems should have increased sensitivity of our test. Another factor that might have affected the influence of sleep is the mild sleep restriction by 1 h in the night before the experiment. However, we have used a similar procedure in a previous study without impairing effects of sleep on memory consolidation (Schönauer et al., 2014b), and even the highly sensitive psychomotor vigilance task is unaffected by 2 × 2 h of sleep loss (Banks and Dinges, 2007).

With the total sample size of *N* = 77, we are able to detect effects above $\eta_{p}^{2}$ = 0.1 with a statistical power of 80% and a significance level of 5% in our experimental design. We can therefore assume that based on the present data it is improbable that an effect of a medium size of sleep on insight problem solving exists. This effect size corresponds to what other studies on cognitive functions of sleep have found (e.g., Schönauer et al., 2014b). For large effects above $\eta_{p}^{2}$ = 0.15, our design had a power of 95%. η^2^ can roughly be interpreted as the percentage of total variance explained by a factor. Analysis of achieved effect sizes $\eta_{p}^{2}$ showed that all relevant effects found explained less than 1% of variance. Effects of that size would require an *N* > 750 to yield significant results with a power of 80%. For small effects of around 1% explained variance, achieved statistical power was 14%. The present experiments therefore cannot exclude the existence of an effect of that size.

We tested the effect of incubation and sleep on solving insight tasks that specifically require a restructuring of the problem representation. During the experiment, we paid careful attention to prevent effects of experimenter expectancy by presenting only standardized written and recorded instructions. Neither sleep nor incubation increased solution rates or the subjective experience of sudden insight. Our study adds to accumulating evidence that sleep does not provide a more suitable environment for problem solving than the wake state. The main function of the reorganization of memory content during sleep might thus lie instead in the extraction of common features of multiple experiences.

## Ethics Statement

This study was carried out in accordance with the recommendations of the Ethics Committee of the Department of Psychology at Ludwig-Maximilians-Universität München with written informed consent from all subjects. All subjects gave written informed consent in accordance with the Declaration of Helsinki. The protocol was approved by the Ethics Committee of the Department of Psychology at Ludwig-Maximilians-Universität München.

## Author Contributions

MS, AD, and SG conceived and designed the experiments. MS and AB performed the research. MS, SB, DP, AB, AD, and SG analyzed the data. MS, SB, DP, and SG wrote the manuscript. All authors discussed the results and revised the paper.

## Conflict of Interest Statement

The authors declare that the research was conducted in the absence of any commercial or financial relationships that could be construed as a potential conflict of interest.
